# Supplementary material for: A Comparative Study Evaluating the Effectiveness of Folate-Based B Vitamin Intervention on Cognitive Function of Older Adults under Mandatory Folic Acid Fortification Policy: A Systematic Review and Meta-Analysis of Randomized Controlled Trials
Source: Nutrients. 2024 Jul 10;16(14):2199. doi: 10.3390/nu16142199 (PMC11279592; doi:10.3390/nu16142199)
Supplement: Supplementary file 1 [file nutrients-16-02199-s001.zip › Figure S1 Risk of bias.pdf]

A

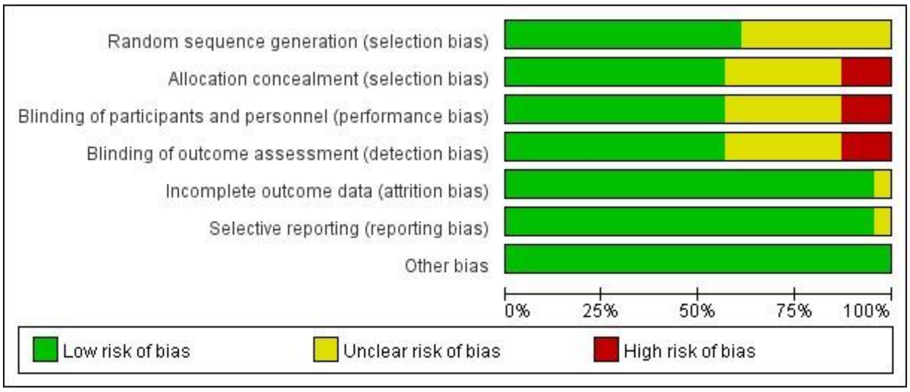

Random sequence generation (selection bias)
Allocation concealment (selection bias)
Blinding of participants and personnel (performance bias)
Blinding of outcome assessment (detection bias)
Incomplete outcome data (attrition bias)
Selective reporting (reporting bias)
Other bias
0%
25%
50%
75%
100%
Low risk of bias
Unclear risk of bias
High risk of bias

B

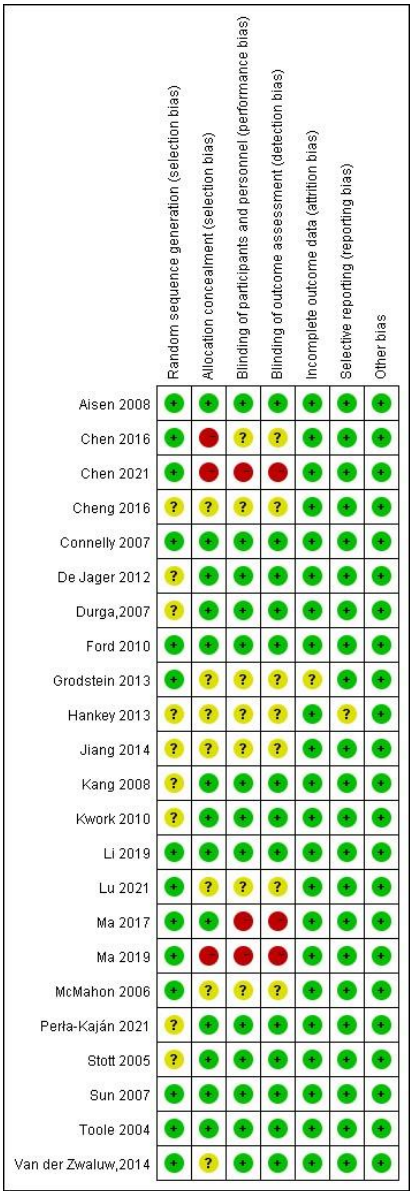

Random sequence generation (selection bias)
Allocation concealment (selection bias)
Blinding of participants and personnel (performance bias)
Blinding of outcome assessment (detection bias)
Incomplete outcome data (attrition bias)
Selective reporting (reporting bias)
Other bias
Aisen 2008
Chen 2016
Chen 2021
Cheng 2016
Connelly 2007
De Jager 2012
Durga,2007
Ford 2010
Grodstein 2013
Hankey 2013
Jiang 2014
Kang 2008
Kwork 2010
Li 2019
Lu 2021
Ma 2017
Ma 2019
McMahon 2006
Perła-Kaján 2021
Stott 2005
Sun 2007
Toole 2004
Van der Zwaluw,2014
