# Supplementary figures and images for: A Comparative Study Evaluating the Effectiveness of Folate-Based B Vitamin Intervention on Cognitive Function of Older Adults under Mandatory Folic Acid Fortification Policy: A Systematic Review and Meta-Analysis of Randomized Controlled Trials
Source: Nutrients. 2024 Jul 10;16(14):2199. doi: 10.3390/nu16142199 (PMC11279592; doi:10.3390/nu16142199)

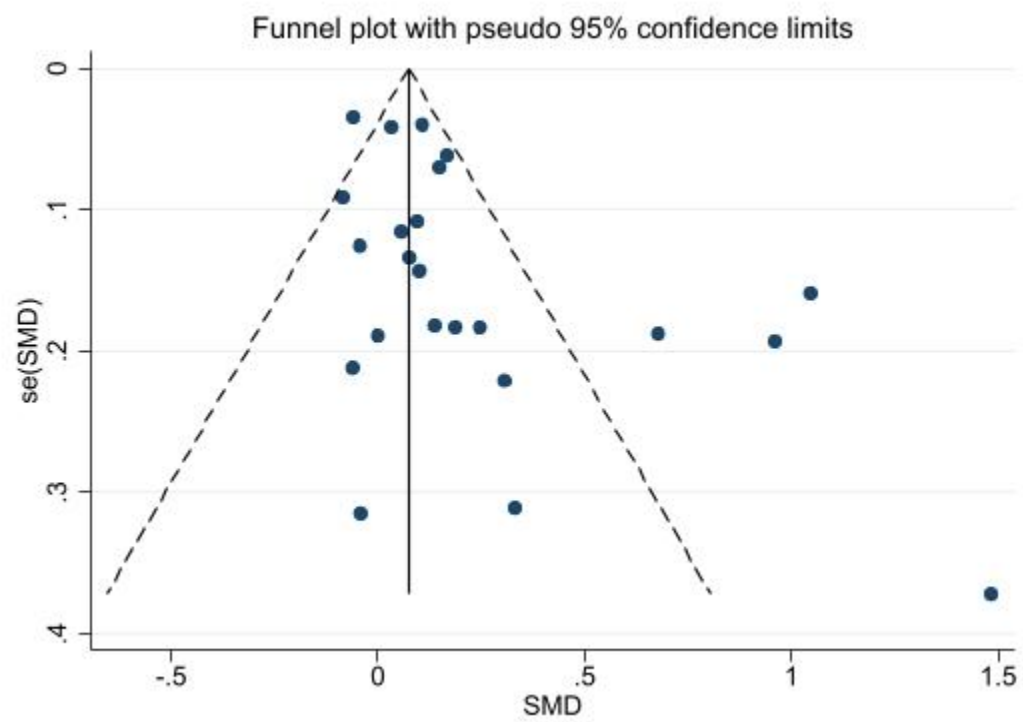

Supplement: Supplementary file 1 [file nutrients-16-02199-s001.zip › Figure S2 Funnel plot global cognition function.pdf]
